# (*E*)-*N*′-(3-Hy­droxy­benzyl­idene)-3-nitro­benzohydrazide

**DOI:** 10.1107/S1600536810049482

**Published:** 2010-12-04

**Authors:** Fu-Lin Mao, Chun-Hua Dai

**Affiliations:** a Department of Chemistry, Yancheng Teachers University, Yancheng 224002, People’s Republic of China

## Abstract

The title compound, C_14_H_11_N_3_O_4_, was prepared by the reaction of 3-nitro­benzohydrazide with 3-hy­droxy­benzalde­hyde. The mol­ecule adopts an *E* configuration about the C=N bond. The dihedral angle between the two benzene rings is 32.3 (2)°. In the crystal, the mol­ecules are linked through inter­molecular N—H⋯O, O—H⋯N, and O—H⋯O hydrogen bonds, forming chains in the *a*-axis direction.

## Related literature

For medical applications of hydrazones, see: Ajani *et al.* (2010 ▶); Zhang *et al.* (2010 ▶); Angelusiu *et al.* (2010 ▶). For related structures, see: Huang & Wu (2010 ▶); Khaledi *et al.* (2010 ▶); Zhou & Yang (2010 ▶); Ji & Lu (2010 ▶); Singh & Singh (2010 ▶); Ahmad *et al.* (2010 ▶). For similar compounds that we have reported recently, see: Dai & Mao (2010*a* ▶,*b* ▶).
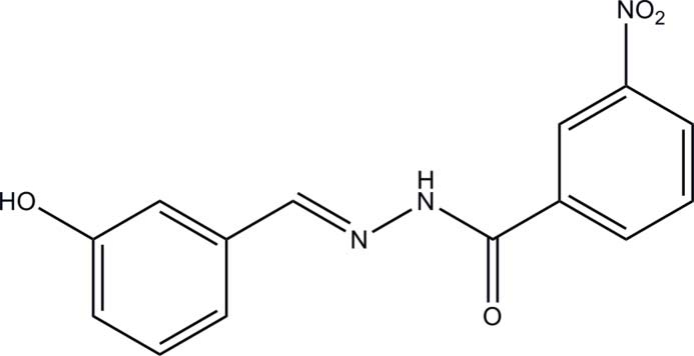


## Experimental

### 

#### Crystal data

C_14_H_11_N_3_O_4_
*M* *_r_* = 285.26
Monoclinic, 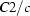

*a* = 16.154 (3) Å
*b* = 13.295 (3) Å
*c* = 13.537 (2) Å
β = 120.324 (2)°
*V* = 2509.5 (8) Å^3^
*Z* = 8
Mo *K*α radiation
μ = 0.11 mm^−1^
*T* = 298 K
0.28 × 0.27 × 0.23 mm

#### Data collection

Bruker SMART CCD area-detector diffractometer
Absorption correction: multi-scan (*SADABS*; Bruker, 2001 ▶) *T*_min_ = 0.969, *T*_max_ = 0.974
6631 measured reflections
2738 independent reflections
1588 reflections with *I* > 2σ(*I*)
*R*_int_ = 0.038

#### Refinement

*R*[*F* ^2^ > 2σ(*F* ^2^)] = 0.054
*wR*(*F* ^2^) = 0.144
*S* = 1.03
2738 reflections
196 parameters
2 restraints
H atoms treated by a mixture of independent and constrained refinement
Δρ_max_ = 0.18 e Å^−3^
Δρ_min_ = −0.25 e Å^−3^

### 

Data collection: *SMART* (Bruker, 2007 ▶); cell refinement: *SAINT* (Bruker, 2007 ▶); data reduction: *SAINT*; program(s) used to solve structure: *SHELXTL* (Sheldrick, 2008 ▶); program(s) used to refine structure: *SHELXTL*; molecular graphics: *SHELXTL*; software used to prepare material for publication: *SHELXTL*.

## Supplementary Material

Crystal structure: contains datablocks global, I. DOI: 10.1107/S1600536810049482/sj5070sup1.cif

Structure factors: contains datablocks I. DOI: 10.1107/S1600536810049482/sj5070Isup2.hkl

Additional supplementary materials:  crystallographic information; 3D view; checkCIF report

## Figures and Tables

**Table 1:** Hydrogen-bond geometry (Å, °)

*D*—H⋯*A*	*D*—H	H⋯*A*	*D*⋯*A*	*D*—H⋯*A*
O1—H1⋯N1^i^	0.85 (1)	2.58 (3)	2.986 (2)	111 (2)
O1—H1⋯O2^i^	0.85 (1)	1.91 (1)	2.758 (2)	174 (3)
N2—H2*A*⋯O4^ii^	0.89 (1)	2.47 (1)	3.354 (3)	171 (2)

Symmetry codes: (i) 
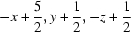
; (ii) 
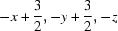
.

**Fig. 1.**
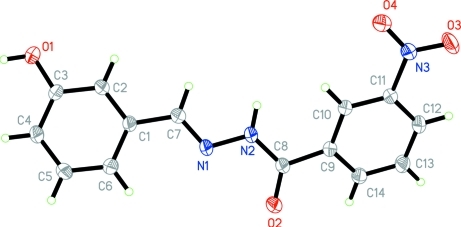
The molecular structure of the title compound showing 30% probability displacement ellipsoids and the atomic numbering.

**Fig. 2.**
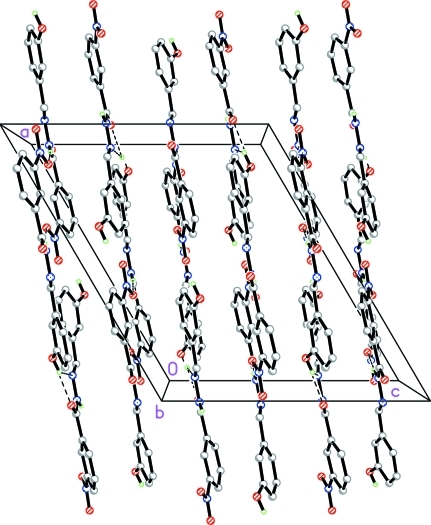
Crystal packing of the title compound, viewed down the b axis. Intermolecular interactions are drawn as dashed lines.

## supplementary crystallographic information

### Comment

In the last few years, medical applications of a number of hydrazone compounds have been received much attention (Ajani *et al.*, 2010; Zhang *et al.*, 2010; Angelusiu *et al.*, 2010). The structures of several hydrazone derivatives have also been determined (Huang & Wu, 2010; Khaledi *et al.*, 2010; Zhou & Yang, 2010; Ji & Lu, 2010; Singh & Singh, 2010; Ahmad *et al.*, 2010). As a continuation of our work on this area (Dai & Mao, 2010*a*,*b*), in this paper, we report the structure of the new derivative *N*'-(3-hydroxybenzylidene)-3-nitrobenzohydrazide.

In the molecule of the title compound, the dihedral angle between the C1···C6 and C9···C14 benzene rings is 32.3 (2)°. The O3/N3/O4 plane forms a dihedral angle of 4.8 (2)° with the C9···C14 benzene ring. The bond lengths and angles are comparable to those found in the hydrazone compounds cited above. In the crystal structure, the hydrazone molecules are linked through intermolecular N—H···O, O–H···N, and O—H···O hydrogen bonds (Table 1), to form one-dimensional chains in the *a* direction, Fig. 2.

### Experimental

The reaction of 3-nitrobenzohydrazide (0.181 g, 1 mmol) with 3-hydroxybenzaldehyde (0.122 g, 1 mmol) in 50 ml methanol at room temperature afforded the title compound. Yellow block-shaped single crystals were formed by slow evaporation of the clear solution in air.

### Refinement

The H1 and H2A atoms were located in a difference Fourier map and refined with N—H = 0.90 (1) Å, O—H = 0.85 (1) Å, and *U*_iso_ = 0.08 Å^2^. Other H atoms were positioned geometrically (C—H = 0.93 Å) and refined as riding with *U*_iso_(H) = 1.2*U*_eq_(C).

### Crystal data

**Table:**

C_14_H_11_N_3_O_4_	*F*(000) = 1184
*M**_r_* = 285.26	*D*_x_ = 1.510 Mg m^−^^3^
Monoclinic, *C*2/*c*	Mo *K*α radiation, λ = 0.71073 Å
Hall symbol: -C 2yc	Cell parameters from 1285 reflections
*a* = 16.154 (3) Å	θ = 2.3–25.0°
*b* = 13.295 (3) Å	µ = 0.11 mm^−^^1^
*c* = 13.537 (2) Å	*T* = 298 K
β = 120.324 (2)°	Block, yellow
*V* = 2509.5 (8) Å^3^	0.28 × 0.27 × 0.23 mm
*Z* = 8	

### Data collection

**Table:**

Bruker SMART CCD area-detector diffractometer	2738 independent reflections
Radiation source: fine-focus sealed tube	1588 reflections with *I* > 2σ(*I*)
graphite	*R*_int_ = 0.038
ω scans	θ_max_ = 27.0°, θ_min_ = 2.1°
Absorption correction: multi-scan (*SADABS*; Bruker, 2001)	*h* = −18→20
*T*_min_ = 0.969, *T*_max_ = 0.974	*k* = −15→16
6631 measured reflections	*l* = −17→17

### Refinement

**Table:**

Refinement on *F*^2^	Primary atom site location: structure-invariant direct methods
Least-squares matrix: full	Secondary atom site location: difference Fourier map
*R*[*F*^2^ > 2σ(*F*^2^)] = 0.054	Hydrogen site location: inferred from neighbouring sites
*wR*(*F*^2^) = 0.144	H atoms treated by a mixture of independent and constrained refinement
*S* = 1.03	*w* = 1/[σ^2^(*F*_o_^2^) + (0.0639*P*)^2^] where *P* = (*F*_o_^2^ + 2*F*_c_^2^)/3
2738 reflections	(Δ/σ)_max_ < 0.001
196 parameters	Δρ_max_ = 0.18 e Å^−^^3^
2 restraints	Δρ_min_ = −0.25 e Å^−^^3^

### Special details

**Table:**

Geometry. All esds (except the esd in the dihedral angle between two l.s. planes) are estimated using the full covariance matrix. The cell esds are taken into account individually in the estimation of esds in distances, angles and torsion angles; correlations between esds in cell parameters are only used when they are defined by crystal symmetry. An approximate (isotropic) treatment of cell esds is used for estimating esds involving l.s. planes.
Refinement. Refinement of F^2^ against ALL reflections. The weighted R-factor wR and goodness of fit S are based on F^2^, conventional R-factors R are based on F, with F set to zero for negative F^2^. The threshold expression of F^2^ > 2sigma(F^2^) is used only for calculating R-factors(gt) etc. and is not relevant to the choice of reflections for refinement. R-factors based on F^2^ are statistically about twice as large as those based on F, and R- factors based on ALL data will be even larger.

### Fractional atomic coordinates and isotropic or equivalent isotropic displacement parameters (Å^2^)

**Table:**

	*x*	*y*	*z*	*U*_iso_*/*U*_eq_	
N1	1.05387 (12)	0.55191 (13)	0.15027 (16)	0.0401 (5)	
N2	0.95666 (13)	0.55067 (14)	0.10982 (17)	0.0422 (5)	
N3	0.59440 (14)	0.63671 (15)	−0.07753 (18)	0.0489 (5)	
O1	1.36619 (12)	0.85288 (13)	0.36202 (19)	0.0695 (6)	
O2	0.95061 (11)	0.38209 (12)	0.09885 (16)	0.0594 (5)	
O3	0.50831 (13)	0.62855 (15)	−0.1151 (2)	0.0883 (8)	
O4	0.63203 (12)	0.71586 (13)	−0.07821 (16)	0.0605 (5)	
C1	1.19663 (15)	0.65018 (16)	0.22714 (18)	0.0371 (5)	
C2	1.23682 (17)	0.74283 (17)	0.2723 (2)	0.0455 (6)	
H2	1.1981	0.7940	0.2736	0.055*	
C3	1.33314 (17)	0.76106 (16)	0.3155 (2)	0.0427 (6)	
C4	1.39012 (16)	0.68658 (17)	0.3115 (2)	0.0458 (6)	
H4	1.4547	0.6986	0.3377	0.055*	
C5	1.35046 (16)	0.59342 (18)	0.2679 (2)	0.0482 (6)	
H5	1.3892	0.5425	0.2662	0.058*	
C6	1.25507 (16)	0.57453 (18)	0.22723 (19)	0.0433 (6)	
H6	1.2299	0.5110	0.1998	0.052*	
C7	1.09381 (16)	0.63653 (17)	0.1815 (2)	0.0435 (6)	
H7	1.0567	0.6922	0.1753	0.052*	
C8	0.91039 (16)	0.46268 (17)	0.08701 (19)	0.0386 (5)	
C9	0.80596 (15)	0.46724 (16)	0.04857 (18)	0.0368 (5)	
C10	0.75046 (15)	0.55308 (16)	0.00497 (18)	0.0385 (5)	
H10	0.7774	0.6130	−0.0008	0.046*	
C11	0.65446 (15)	0.54756 (16)	−0.02961 (19)	0.0383 (5)	
C12	0.61190 (17)	0.46063 (17)	−0.0223 (2)	0.0458 (6)	
H12	0.5469	0.4590	−0.0461	0.055*	
C13	0.66761 (17)	0.37664 (18)	0.0209 (2)	0.0501 (6)	
H13	0.6403	0.3169	0.0264	0.060*	
C14	0.76350 (16)	0.37974 (17)	0.0561 (2)	0.0444 (6)	
H14	0.8005	0.3220	0.0856	0.053*	
H2A	0.9284 (18)	0.6098 (12)	0.104 (2)	0.080*	
H1	1.4208 (11)	0.863 (2)	0.369 (2)	0.080*	

### Atomic displacement parameters (Å^2^)

**Table:**

	*U*^11^	*U*^22^	*U*^33^	*U*^12^	*U*^13^	*U*^23^
N1	0.0246 (10)	0.0399 (11)	0.0500 (11)	0.0010 (8)	0.0146 (9)	0.0013 (9)
N2	0.0263 (11)	0.0385 (11)	0.0560 (12)	0.0005 (8)	0.0166 (10)	−0.0001 (9)
N3	0.0351 (12)	0.0409 (12)	0.0686 (14)	0.0015 (9)	0.0245 (11)	−0.0006 (10)
O1	0.0477 (12)	0.0428 (10)	0.1195 (16)	−0.0136 (9)	0.0433 (12)	−0.0173 (10)
O2	0.0378 (10)	0.0387 (10)	0.0931 (14)	0.0057 (8)	0.0267 (10)	0.0032 (9)
O3	0.0312 (11)	0.0640 (13)	0.151 (2)	0.0091 (9)	0.0322 (13)	0.0204 (12)
O4	0.0478 (11)	0.0405 (10)	0.0916 (14)	0.0029 (8)	0.0340 (11)	0.0033 (9)
C1	0.0282 (12)	0.0364 (12)	0.0427 (13)	−0.0002 (10)	0.0150 (11)	0.0027 (10)
C2	0.0370 (14)	0.0357 (13)	0.0662 (16)	0.0035 (10)	0.0279 (13)	0.0048 (11)
C3	0.0364 (14)	0.0335 (12)	0.0550 (15)	−0.0060 (10)	0.0208 (12)	−0.0018 (11)
C4	0.0274 (13)	0.0472 (15)	0.0556 (15)	−0.0001 (11)	0.0157 (12)	0.0010 (12)
C5	0.0363 (14)	0.0464 (15)	0.0580 (16)	0.0055 (11)	0.0210 (13)	−0.0037 (12)
C6	0.0358 (14)	0.0396 (13)	0.0488 (15)	−0.0033 (10)	0.0171 (12)	−0.0062 (10)
C7	0.0347 (13)	0.0345 (13)	0.0579 (15)	0.0033 (10)	0.0208 (12)	0.0025 (11)
C8	0.0299 (13)	0.0370 (13)	0.0438 (13)	0.0017 (10)	0.0149 (11)	0.0019 (10)
C9	0.0305 (12)	0.0370 (12)	0.0392 (13)	−0.0017 (10)	0.0148 (11)	−0.0035 (10)
C10	0.0318 (13)	0.0341 (12)	0.0473 (14)	−0.0022 (9)	0.0183 (11)	−0.0024 (10)
C11	0.0307 (13)	0.0369 (12)	0.0444 (13)	0.0010 (10)	0.0168 (11)	−0.0037 (10)
C12	0.0316 (13)	0.0480 (14)	0.0556 (16)	−0.0052 (11)	0.0205 (12)	−0.0018 (12)
C13	0.0389 (15)	0.0419 (14)	0.0668 (17)	−0.0062 (11)	0.0247 (13)	0.0061 (12)
C14	0.0373 (14)	0.0363 (13)	0.0524 (15)	0.0009 (10)	0.0173 (12)	0.0049 (11)

### Geometric parameters (Å, °)

**Table:**

N1—C7	1.259 (3)	C4—H4	0.9300
N1—N2	1.378 (2)	C5—C6	1.372 (3)
N2—C8	1.338 (3)	C5—H5	0.9300
N2—H2A	0.892 (10)	C6—H6	0.9300
N3—O4	1.218 (2)	C7—H7	0.9300
N3—O3	1.221 (2)	C8—C9	1.495 (3)
N3—C11	1.460 (3)	C9—C14	1.380 (3)
O1—C3	1.354 (3)	C9—C10	1.386 (3)
O1—H1	0.850 (10)	C10—C11	1.377 (3)
O2—C8	1.221 (2)	C10—H10	0.9300
C1—C6	1.379 (3)	C11—C12	1.374 (3)
C1—C2	1.383 (3)	C12—C13	1.366 (3)
C1—C7	1.462 (3)	C12—H12	0.9300
C2—C3	1.379 (3)	C13—C14	1.372 (3)
C2—H2	0.9300	C13—H13	0.9300
C3—C4	1.372 (3)	C14—H14	0.9300
C4—C5	1.382 (3)		
			
C7—N1—N2	115.61 (18)	C1—C6—H6	120.1
C8—N2—N1	119.61 (18)	N1—C7—C1	122.2 (2)
C8—N2—H2A	123.3 (19)	N1—C7—H7	118.9
N1—N2—H2A	117.0 (19)	C1—C7—H7	118.9
O4—N3—O3	122.8 (2)	O2—C8—N2	122.6 (2)
O4—N3—C11	118.86 (19)	O2—C8—C9	120.9 (2)
O3—N3—C11	118.3 (2)	N2—C8—C9	116.47 (19)
C3—O1—H1	110 (2)	C14—C9—C10	119.1 (2)
C6—C1—C2	118.7 (2)	C14—C9—C8	117.1 (2)
C6—C1—C7	122.8 (2)	C10—C9—C8	123.7 (2)
C2—C1—C7	118.5 (2)	C11—C10—C9	118.4 (2)
C3—C2—C1	121.4 (2)	C11—C10—H10	120.8
C3—C2—H2	119.3	C9—C10—H10	120.8
C1—C2—H2	119.3	C12—C11—C10	122.6 (2)
O1—C3—C4	123.7 (2)	C12—C11—N3	118.1 (2)
O1—C3—C2	116.8 (2)	C10—C11—N3	119.30 (19)
C4—C3—C2	119.5 (2)	C13—C12—C11	118.2 (2)
C3—C4—C5	119.2 (2)	C13—C12—H12	120.9
C3—C4—H4	120.4	C11—C12—H12	120.9
C5—C4—H4	120.4	C12—C13—C14	120.5 (2)
C6—C5—C4	121.3 (2)	C12—C13—H13	119.7
C6—C5—H5	119.3	C14—C13—H13	119.7
C4—C5—H5	119.3	C13—C14—C9	121.0 (2)
C5—C6—C1	119.8 (2)	C13—C14—H14	119.5
C5—C6—H6	120.1	C9—C14—H14	119.5

### Hydrogen-bond geometry (Å, °)

**Table:**

*D*—H···*A*	*D*—H	H···*A*	*D*···*A*	*D*—H···*A*
O1—H1···N1^i^	0.85 (1)	2.58 (3)	2.986 (2)	111 (2)
O1—H1···O2^i^	0.85 (1)	1.91 (1)	2.758 (2)	174 (3)
N2—H2A···O4^ii^	0.89 (1)	2.47 (1)	3.354 (3)	171 (2)

Symmetry codes: (i) −*x*+5/2, *y*+1/2, −*z*+1/2; (ii) −*x*+3/2, −*y*+3/2, −*z*.
